# Accelerated innervation of biofabricated skeletal muscle implants containing a neurotrophic factor delivery system

**DOI:** 10.3389/fbioe.2024.1476370

**Published:** 2024-10-28

**Authors:** Vladimir Mashanov, Erika Billman, Aurelia Poerio, Mary Kaufmann, Dehui Lai, J. William Vaughan, Ickhee Kim, Young Min Ju, Anthony Atala, James J. Yoo, Ji Hyun Kim

**Affiliations:** ^1^ Wake Forest Institute for Regenerative Medicine, Wake Forest University School of Medicine, Winston-Salem, NC, United States; ^2^ Institut Jean Lamour, Université de Lorraine, Nancy, France; ^3^ School of Medicine, University of North Carolina at Chapel Hill, Chapel Hill, NC, United States; ^4^ Department of Urology, Fifth Affiliated Hospital of Guangzhou Medical University, Guangzhou, China

**Keywords:** bioengineered skeletal muscle, innervation, neurotrophic factors, CNTF, GDNF, controlled-release delivery system

## Abstract

**Introduction:**

Volumetric muscle loss (VML) is one of the most severe and debilitating conditions in orthopedic and regenerative medicine. Current treatment modalities often fail to restore the normal structure and function of the damaged skeletal muscle. Bioengineered tissue constructs using the patient’s own cells have emerged as a promising alternative treatment option, showing positive outcomes in fostering new muscle tissue formation. However, achieving timely and proper innervation of the implanted muscle constructs remains a significant challenge. In this study, we present a clinically relevant strategy aimed at enhancing and sustaining the natural regenerative response of peripheral nerves to accelerate the innervation of biofabricated skeletal muscle implants.

**Methods:**

We previously developed a controlled-release neurotrophic factor delivery system using poly (lactic-co-glycolic acid) (PLGA) microspheres encapsulating ciliary neurotrophic factor (CNTF) and glial cell line-derived neurotrophic factor (GDNF). Here, we incorporate this neurotrophic factor delivery system into bioprinted muscle constructs to facilitate innervation *in vivo*.

**Results:**

Our results demonstrate that the neurotrophic factors released from the microspheres provide a chemical cue, significantly enhancing the neurite sprouting and functional innervation of the muscle cells in the biofabricated muscle construct within 12 weeks post-implantation.

**Discussion:**

Our approach provides a clinically applicable treatment option for VML through accelerated innervation of biomanufactured muscle implants and subsequent improvements in functionality.

## 1 Introduction

Traumatic or surgical loss of large volumes of skeletal muscle tissue, known as volumetric muscle loss (VML), results in permanent extensive defects due to the human body’s limited inherent regenerative capacities (Grogan and Hsu, 2011). The loss of the muscle tissue is further exacerbated by damage to the nervous system, leading to degeneration of denervated muscle, drastically reducing the muscle function and quality of life (Turner and Badylak, 2012; Ahuja et al., 2021). To restore the lost function in human patients, an effective intervention therapy needs to be developed.

The current standard of care for patients with VML involves autologous muscle transplant to the site of the injury (Turner and Badylak, 2012; Mertens et al., 2014; Grogan and Hsu, 2011). However, this approach primarily focuses on bone coverage and infection prevention, rather than on complete restoration of muscle function (Grogan and Hsu, 2011; Ahuja et al., 2021). The limitations of this approach include high variability in outcomes among the patients, disruption of anatomy and biomechanics in both donor and recipient sites, limited donor tissue supply, donor site morbidity, prolonged denervation of muscle implants, and potential graft failure due to infection or necrosis (Grogan and Hsu, 2011; Turner and Badylak, 2012; Ahuja et al., 2021). These limitations significantly reduce treatment efficacy in achieving a full return to pre-injury function (Mertens et al., 2014).

Given that repairing large VML defects surpasses the body’s natural regenerative abilities, a tissue engineering-based therapeutic approach using culture-expanded autologous muscle cells can be a promising effective treatment option (Mertens et al., 2014). While efforts have been made to develop more efficient therapeutic approaches for replenishing lost muscle fibers, addressing proper innervation of the muscle tissue remains a critical challenge in the clinical treatment of VML (Grogan and Hsu, 2011; Das et al., 2020; Ahuja et al., 2021). Insufficient or delayed innervation can lead to atrophy of the regenerated or implanted muscle tissue (Turner and Badylak, 2012).

One widely used clinical intervention for addressing muscle innervation issues involves autologous muscle transplantation with neurorrhaphy. These procedures, however, often lead to complications such as poor regeneration and extensive scarring (Ahuja et al., 2021). Moreover, surgical repair alone may not be sufficient, as regenerating neurites often fail to properly innervate muscle fibers (Gordon, 2020). Another strategy to address the innervation challenge is pre-innervation by incorporating neurons into the biofabricated muscle implant (Kim et al., 2020; Das et al., 2020). This approach has shown significant improvements in muscle fiber differentiation and long-term survival. Nevertheless, its clinical applicability may be limited, due to the direct integration of neuronal cell bodies into the tissue-engineered muscle. This integration fails to recapitulate the normal anatomical and functional relationships between neural and muscular components. Furthermore, manufacturing such composite implants would require harvesting motoneurons or neural stem cells from the patient’s central nervous system, potentially introducing complications and risks that outweigh the benefits (Gordon, 2020; Ahuja et al., 2021).

Our study aims to develop a clinically relevant approach to enhance the innervation of tissue-engineered skeletal muscle implants by leveraging the natural regenerative response of the host peripheral nervous system. We hypothesize that establishing proper innervation involves two key strategies: (a) enhancing the growth and sprouting of the host neurites into the implanted construct and (b) promoting new motor endplates (neuromuscular junctions) formation on muscle cells within the construct. Our previous studies have demonstrated that the second objective can be achieved via the pre-formation of acetylcholine receptor (AChR) clusters on muscle cells in biomanufactured constructs by treating the latter with proteoglycan agrin prior to implantation (Ko et al., 2013; Gilbert-Honick et al., 2020; Kim et al., 2021). However, effectively directing and accelerating the growth of the peripheral axons to the muscle cells within the implant has remained a challenge.

We previously demonstrated that two neurotrophic factors, CNTF (ciliary neurotrophic factor) and GDNF (glial cell line-derived neurotrophic factor), act synergistically as a morphogenic cue to induce directed growth of peripheral neurites towards the source of their gradient (Mashanov et al., 2020). Both these factors are naturally released by Schwann cells and other cell types (Richardson, 1994; Lie and Weis, 1998; Zigmond, 2012; Duarte Azevedo et al., 2020), but this intrinsic expression is not sufficient for sustaining full regeneration and innervation (Boyd and Gordon, 2003). Moreover, as morphogens, the source of their gradient needs to be located within the skeletal muscle implant to induce a proper guidance response. We thus developed a controlled neurotrophic factor delivery system using CNTF and GDNF-loaded poly (lactic-co-glycolic acid) (PLGA) microspheres (Poerio et al., 2022) that ensures a sustained release of these factors in physiologically relevant concentrations, enhancing neurite sprouting from ganglia *in vitro* (Poerio et al., 2022). This vehicle is compatible with our previously established skeletal muscle tissue engineering protocols for three-dimensional (3D) bioprinting of skeletal muscle implants (Kang et al., 2016; Kim et al., 2018).

The main objective of the present study is to investigate the feasibility of this neurotrophic factor delivery system in directing neuronal growth toward the skeletal muscle implant and accelerating innervation *in vivo*. To this end, we incorporated the CNTF/GDNF-loaded PLGA microspheres into the bioprinted muscle constructs and evaluated their innervation potential in both *in vitro* and *in vivo* settings. We demonstrated that bioprinted skeletal muscle constructs containing the neurotrophic factor delivery system induced extensive peripheral neurite sprouting *in vitro* and *in vivo*. In an *in vivo* transposed nerve implantation model (Ko et al., 2013; Kang et al., 2016), the constructs with CNTF/GDNF-loaded microspheres exhibited significantly higher levels of sustained innervation compared to constructs with freely dissolved neurotrophic factors without microspheres.

## 2 Materials and methods

### 2.1 Preparation of CNTF/GDNF-loaded PLGA microspheres

The PLGA microspheres loaded with CNTF and GDNF were prepared using a standard water-in-oil-in-water double emulsification protocol, as previously described (Poerio et al., 2022). Briefly, commercially available recombinant human CNTF (R&D, 257-NT) and GDNF (Millipore Sigma, GF322) were reconstituted as per the manufacturers’ instructions and combined at a concentration of 1 mg/mL in PBS containing 0.1% (w/v) bovine serum albumin (BSA). This solution of neurotrophic factors was then added to a 10% (w/v) PLGA (50:50, Lactel, B6010-2) solution in chloroform at a ratio of 1:100 (v/v) and emulsified for 30 s on ice with a Vibra CellVCX 400 ultrasonicator (Sonics & Materials Inc.) set at a 13% amplitude. The resulting first water-in-oil emulsion was immediately added to 3% (v/w) polyvinyl alcohol (PVA, Sigma, 341584) at a 1:100 (v/v) ratio and vortexed at a maximum speed using a vortex mixer (Fisher, 02215414). This final water-in-oil-in-water emulsion was then stirred for 4 h at room temperature to allow for chloroform evaporation. The resulting mixture was filtered through a 100 μ m filter, and the microspheres were collected by centrifugation, washed three times with deionized water, freeze-dried, and stored at −20 ° C until needed. The PLGA microspheres produced using this protocol contained 0.1 μ g/mg of CNTF and GDNF. As previously reported, this approach yields spherical microspheres with a median diameter of 23.4 μ m and a size distribution range of 1.8–44.6 μ m (Poerio et al., 2022).

### 2.2 Expansion of human skeletal muscle progenitor cells (hMPCs) for bioprinting

Patient-derived hMPCs were isolated from muscle biopsies by the Translational Core at the Wake Forest Institute for Regenerative Medicine, as described elsewhere (Eberli et al., 2009; Kim et al., 2020; Kim et al., 2021). Briefly, the tissue samples were washed in 40 mg/mL gentamicin solution, minced into small ( ≤ 1 mm^3^) fragments, and enzymatically digested with collagenase HA (12 Wünsch Units/mL, VitaCyte) and BP protease (33,000 U/mL, VitaCyte) for 45 min at 37 ° C. The digested tissue was then vigorously homogenized by pipetting, and the resulting suspension was passed through a 100 μ m filter. The cells were collected by centrifugation at 300 × g for 5 min and plated on a collagen type I dish for 20–24 h for fibroblast depletion. The cells were then expanded, cryopreserved and stored in liquid nitrogen until needed for the experiments. The banked cells were at least 25% positive for the myogenic transcription factor MyoD and the early-stage myogenesis marker desmin, while being less than 10% positive for myosin heavy chain, a late muscle differentiation marker.

For bioprinting, the cells were thawed and expanded up to passage five in a growth medium (DMEM/High Glucose with 20% (v/v) fetal bovine serum, 2% (w/v) chick embryo extract, and 1% (v/v) penicillin/streptomycin).

### 2.3 Biofabrication of 3D bioprinted skeletal muscle constructs

**FIGURE 1:**
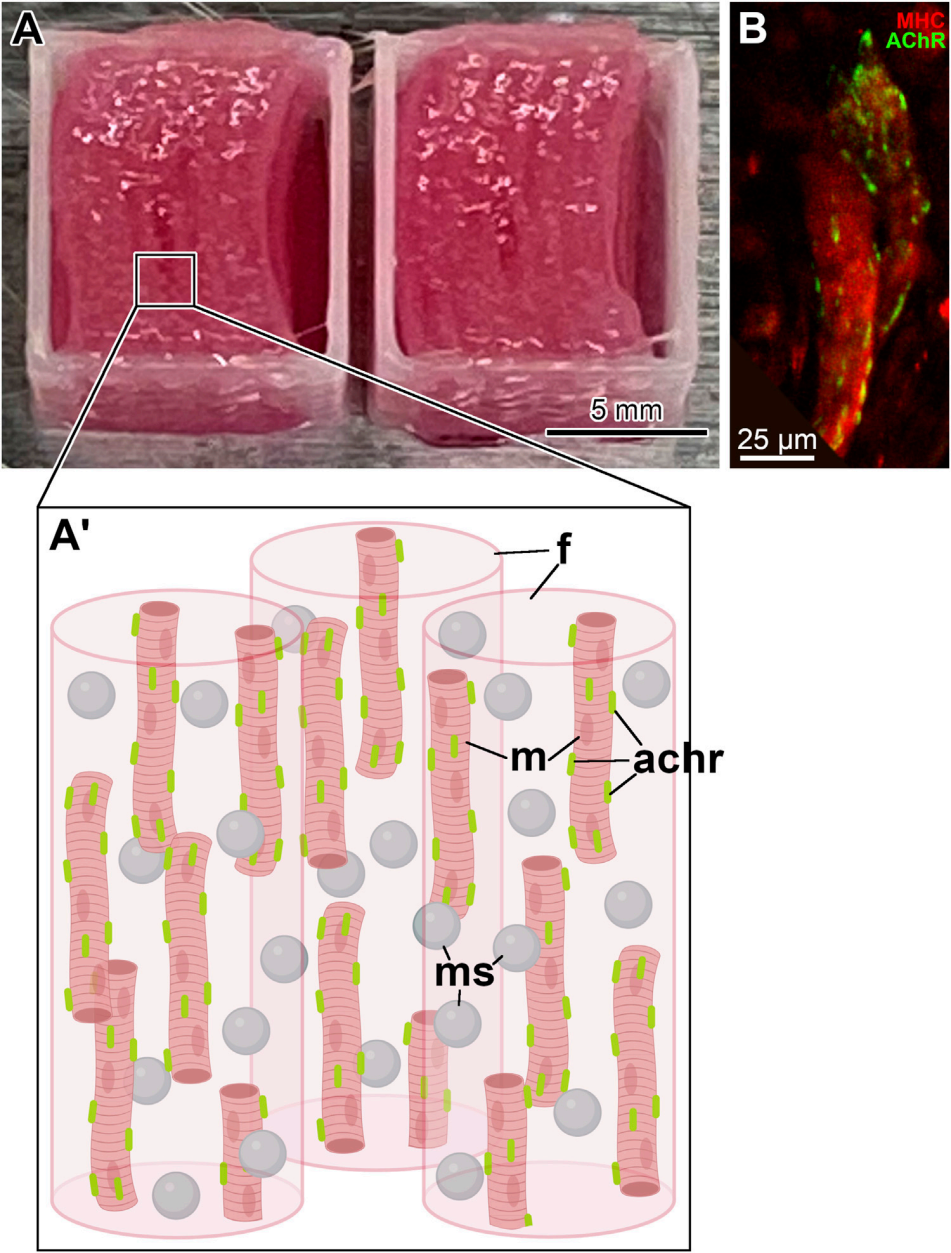
Bioprinted skeletal muscle constructs. **(A)** A pair of fully printed skeletal muscle constructs with a neurotrophic factor delivery system. They have dimensions of 10 mm × 10 mm × 4 mm (W × L × H) and are composed of eight layers of cell-laden fibrinogen-based bioink with sacrificial bioink spacers. The cell-laden bioink contains CNTF/GDNF-loaded microspheres. In addition, an anchoring structure composed of PCL is deposited as a square frame at the periphery of the implant. **(A′)** Diagram of the microscale organization of the muscle construct. Created with BioRender. Abbreviations: *achr*–AChR cluster; *f* – fibrin hydrogel; *m*–muscle cell; *ms*–microsphere. **(B)** Differentiated muscle cells *(red)* with induced pre-formed AChR clusters *(green)* in the muscle construct prior to implantation. The sample was immunostained with antibodies against myosin heavy chain (MHC, red) and AChR (green). The image is a maximum intensity Z-projection of a confocal stack.

The skeletal muscle constructs (Figure 1) were biofabricated using the 3D Integrated Tissue-Organ Printing (ITOP) system (Supplementary Figure S1) developed at the Wake Forest Institute for Regenerative Medicine Kang et al. (2016). Extrusion bioprinting was performed as previously described (Kim et al., 2018; Poerio et al., 2022). The protocol involved three components: (a) a fibrinogen-based bioink containing hMPCs, (b) a gelatin-based sacrificial bioink, and (c) the supporting pillar structure (Supplementary Figure S1).

The fibrinogen-based bioink was prepared by dissolving 20 mg/mL fibrinogen (Sigma, F8630), 35 mg/mL gelatin (Sigma, G6144), and 3 mg/mL hyaluronic acid (Sigma, 53747) in DMEM/High Glucose containing 10% (v/v) glycerol. This bioink carried hMPCs ( $3\times 10^{7}$ cells/mL) with or without the extrinsic neurotrophic factors (CNTF and GDNF), which were either added directly or encapsulated in the PLGA microspheres (see below).

The gelatin-based sacrificial bioink had the same composition as above, but did not contain fibrinogen, and was never loaded with cells or neurotrophic factors. During the layer-by-layer bioprinting process, filaments of the sacrificial hydrogel alternate with and separate strands of the cell-laden fibrin hydrogel (Supplementary Figure S1). Rapid dissolution of the sacrificial gelatin during the post-biofabrication culturing at 37 ° C creates an ordered system of microchannels in the bioprinted construct, facilitating nutrient diffusion, gas exchange, and, ultimately, cell viability throughout the inner core of the structure (Kim et al., 2018).

The supporting pillar structure was composed of polycaprolactone (PCL, MW 43,000–50,000, Polysciences), and each printed layer was deposited as a square frame on the outside perimeter of the construct (Figure 1; Supplementary Figure S1). It served to anchor the filaments of the cell-laden fibrin hydrogel and maintained the mechanical stability of the bioprinted construct (Kim et al., 2018; Poerio et al., 2022). Importantly, the anchoring system also allowed the muscle cells within the construct to align orderly and uniaxially (Kim et al., 2018).

For *in vivo* implantation, the fully printed skeletal muscle constructs (Figures 1A, A’) were composed of eight stacked layers of cell-laden bioink with sacrificial bioink spacers and had dimensions of 10×10 × 4 mm^3^. For the *in vitro* neurite outgrowth assay (see below), the thickness of the constructs was reduced to two layers to facilitate microscopic imaging. The bioprinted constructs were immediately crosslinked with 20 UI/mL thrombin (Sigma, T4648) for 30–60 min at room temperature, transferred into 12-well plates, and cultured in the growth medium overnight.

Differentiation of hMPCs in the bioprinted muscle constructs was induced by culturing them in a differentiation medium containing DMEM/High Glucose with 2% (v/v) horse serum, 1% (v/v) Insulin-Transferrin-Selenium (ITS), 250 nm dexamethasone, and 1% (v/v) penicillin/streptomycin (Kim et al., 2018). Furthermore, the pre-formation of AChR clusters on the sarcolemma of differentiated hMPCs in all cellularized constructs was induced by supplementing the differentiation medium with the nerve-derived heparan sulfate proteoglycan clustering factor agrin at 150 ng/mL (Bruneau et al., 2005; Ko et al., 2013) (Figure 1B).

### 2.4 *In vitro* neurite outgrowth assay

To evaluate whether the microsphere-based neurotrophic factor delivery system incorporated into bioprinted muscle constructs can facilitate neurite outgrowth, we implemented the following *in vitro* neurite outgrowth assay. Three different types of constructs were produced, all containing $3\times 10^{7}$ hMPCs per mL of fibrin-based cell-laden hydrogels but differing in whether or not they contained extrinsic CNTF and GDNF:

1. *Control*–The constructs containing no extrinsic neurotrophic factors and no microspheres;
2. *NFs*–The constructs containing CNTF and GDNF directly mixed into the fibrinogen-based bioink at 0.5 μ g/mL each;
3. *MSs*–The constructs containing CNTF/GDNF-loaded PLGA microspheres with the overall load of the neurotrophic factors matching those in group 2 *(NFs).*

Chick embryo dorsal root ganglia (DRGs) were used as a source of growing neurites. They were extracted from stage 33 ( ∼ E8) chick embryos and collected in the low serum culture medium composed of DMEM/High Glucose with 2% (v/v) horse serum and 1% (v/v) penicillin/streptomycin (Mashanov et al., 2020). The DRGs were then positioned on top of the skeletal muscle constructs, one per construct, and cultured for 2, 7, and 14 days. The medium was changed every other day.

The samples were immunostained, imaged, and quantified as previously described (Mashanov et al., 2020; Poerio et al., 2022). Briefly, they were fixed in 10% (v/v) neutral buffered formalin with 0.1% (v/v) Triton X-100 overnight at 4 ° C, washed in PBS and blocked in the Protein Block solution (Dako, X0909). The anti-neurofilament antibodies (Abcam, AB4680) were applied overnight at 4 ° C at a dilution of 1:1,000. After extensive washes with PBS, the Alexa Fluor 488-conjugated antibodies (ThermoFisher, A-11039) were applied overnight at 4 ° C at a 1:200 dilution. All antibody dilutions were prepared in the Antibody Diluent (Dako, D3022). After the final washes, the samples were mounted in the Vectashield medium with DAPI (Vector Laboratories, H-1200) diluted with PBS at a ratio of 1:1. The samples were imaged with a Leica TCS LSI macro confocal microscope.

**FIGURE 2:**
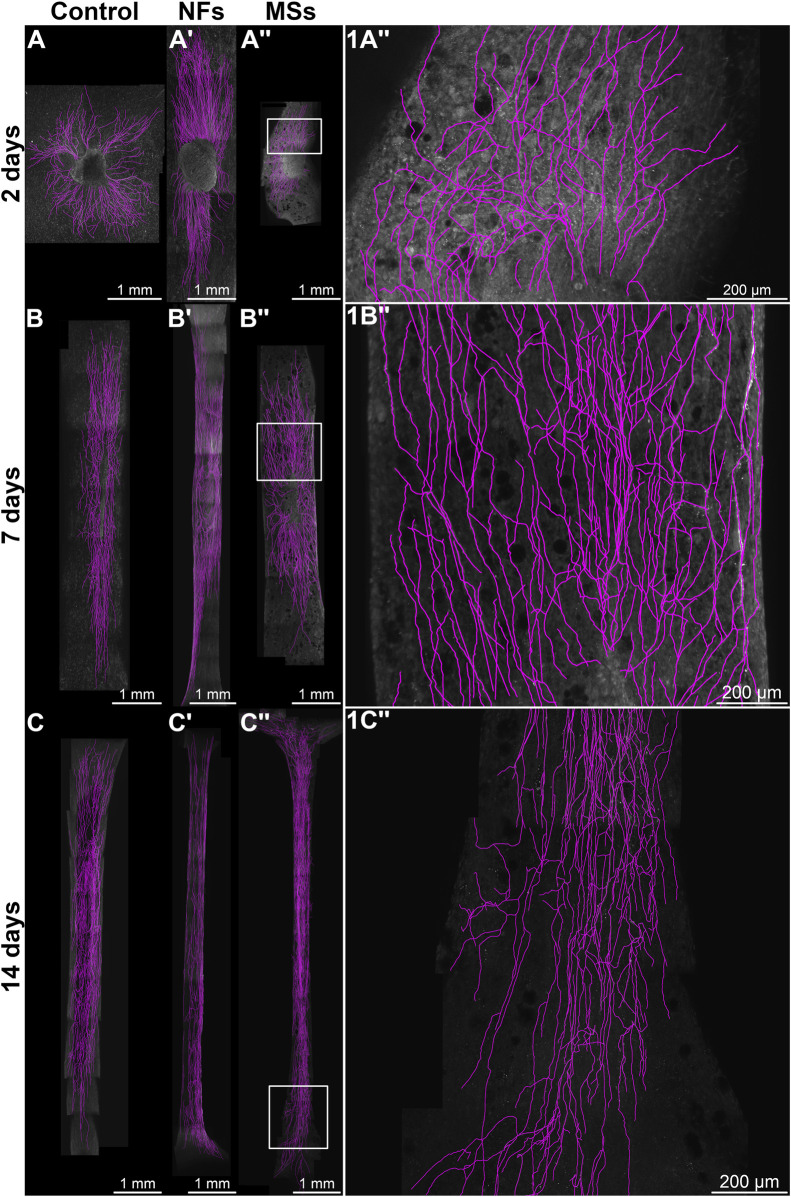
Representative micrographs of chick embryo dorsal root ganglia (DRGs) cultured on 3D bioprinted skeletal muscle constructs in the *in vitro* neurite outgrowth assay. In the *Control* group **(A–C)**, the constructs contained no neurotrophic factors. The microsphere-free constructs in the *NFs* group **(A′–C′)** contained CNTF and GDNF directly mixed into the hydrogel of the construct. The constructs in the *MSs* group **(A′′–C′′)** contained the matching load of the neurotrophic factors encapsulated in PLGA microspheres. The ganglia were cultured on the muscle constructs for 2 days **(A, A′′)**, 7 days **(B, B′′)**, and 14 days **(C, C′′)**. Micrographs in the right column **(1A′′–1C′′)** are representative high-magnification images [of the boxed areas in **(A′′–C′′)**, respectively]. The samples were then fixed and immunostained with anti-neurofilament antibodies. All images are maximum intensity Z-projections of confocal stacks. The neurites were traced with the NeuronJ plugin in the Fiji/ImageJ software and are shown in magenta.

Neurite outgrowth was quantified on maximum intensity Z-projections of confocal stacks using the NeuronJ plugin (Meijering et al., 2004) in the Fiji/ImageJ software (Schindelin et al., 2012) (Figure 2). Three neurite growth metrics were calculated as previously described (Mashanov et al., 2020): the number of neurites sprouting from each ganglion; the total neurite outgrowth per ganglion (i.e., the total length of all neurites per ganglion); and the average neurite length.

### 2.5 *In vivo* transposed nerve assay

To evaluate the capacity of the microsphere-based neurotrophic factor delivery system to facilitate the innervation of bioprinted skeletal muscle constructs in *vivo* settings, we conducted a transposed nerve assay in a rat model. The full-size eight-layer skeletal muscle implants were bioprinted as described above. Four experimental groups were involved:

1. *Acellular*–Bioprinted skeletal muscle constructs without cells and extrinsic neurotrophic factors.
2. *Control*–Cellularized bioprinted skeletal muscle constructs without extrinsic neurotrophic factors containing hMPCs ( $30\times 10^{6}$ cells per mL of bioink) with pre-formed AChR clusters.
3. *NFs*–Cellularized bioprinted microsphere-free muscle constructs with neurotrophic factors (CNTF and GDNF, 0.5 μ g/mL each) directly dissolved in the bioink.
4. *MSs*–Cellularized bioprinted muscle constructs with CNTF/GDNF-loaded PLGA microspheres with the total amount of the neurotrophic factors matching that in the *NFs* cohort.

In all four experimental cohorts, the constructs were wrapped around the distal end of the dissected common peroneal nerve (CPN) and implanted under the fascia of the right gluteus muscle of athymic male rats (body weight 250–300 g, Charles River Laboratory, Wilmington, MA) (Supplementary Figure S2) (Ko et al., 2013; Kang et al., 2016; Kim et al., 2021). Electrophysiological assessment and tissue harvesting were performed at 4 weeks, 8 weeks, and 12 weeks post-implantation (n = 4 per group/time point). All procedures were performed following a protocol approved by the Institutional Animal Care and Use Committee (IACUC) at Wake Forest University.

For the electrophysiological assessment of the functional integration of the transposed nerve into the implanted muscle construct, the transplanted CPN was stimulated at 2 mA, and the amplitude of the compound muscle action potential (CMAP) from the implanted area was recorded using a Sierra Wave electromyography (EMG)/nerve conduction velocity (NCV) instrument (Cadwell).

After collecting the electrophysiological data, tissue samples, including the nerve, the implant, and the adjacent host muscle tissue, were harvested and processed for histological and immunohistochemical evaluation. The specimens were snap-frozen in liquid nitrogen and embedded in the Tissue-Tek O.C.T. Compound (Sakura). Serial cryosections (10 μ m thick) were cut using a Leica CM1860 cryostat and collected on Apex Superior Adhesive Slides (Leica). For histological evaluation, every 15th slide in the series was stained with hematoxylin and eosin (H&E) to assess the microanatomical organization of the specimens and to select sections for immunostaining and quantitative analysis.

For immunohistochemical staining, the slides were washed with PBS for 10 min and then fixed with 10% (v/v) neutral buffered formalin containing 0.1% (v/v) Triton X-100 for 10 min at room temperature. They were then washed again in PBS ( 3×10 min) and postfixed in ice-cold methanol for 10 min at −20 ° C. After another wash in PBS ( 3×10 min), the sections were blocked in the Blocking Solution (Dako, X0909). Primary antibodies were applied at 4 ° C overnight (Supplementary Table S1). After washing off the unbound primary antibodies in PBS, secondary antibodies (Supplementary Table S1) were applied for 1 h at room temperature. After the final round of PBS washes, the slides were coverslipped in the Vectashield mounting medium with DAPI (Vector Laboratories, H-1200). The sections were photographed with an Olympus BX63 motorized compound microscope.

The effect of the extrinsic neurotrophic factors on the innervation of the implanted construct was quantitatively assessed in cryosections immunostained with an anti-neurofilament antibody. At least six non-consecutive sections per individual were quantified using Fiji/ImageJ (Schindelin et al., 2012). Two metrics were employed: (a) the number of neurites per mm^2^ of the implant area and (b) the cumulative length of the sprouting neurites per mm^2^ of the implant area.

### 2.6 *In vitro* hMPC growth/viability and differentiation assay

To assess the effect of the extrinsic CNTF and GDNF on the hMPC growth, viability, and differentiation, an *in vitro* assay comprising four experimental cohorts: (a) *Control*, receiving no neurotrophic factors; (b) *CNTF-only* (0.5 μ g/mL); (c) *GNDF-only* (0.5 μ g/mL); and (d) *CNTF + GDNF* (0.5 μ g/mL each) was implemented. The experimental parameters, including time in culture, base medium composition, and concentration of CNTF/GDNF, matched those for the biofabrication of the muscle constructs, as described above.

For the growth/viability assay, the hMPCs were seeded in 48-well plates in the growth medium at 2,500 cells/well. After 24 h, the medium was replaced with the differentiation medium containing 150 ng/mL agrin with or without neurotrophic factors depending on the treatment cohort. The medium was then changed after 2, 4, and 7 days. To assess hMPCs growth/viability, the alamarBlue assay (ThermoFisher, DAL1100) was performed on days 0 (2 h after seeding), 3, 5, and 8 following the manufacturer’s protocol (n = 6 per group/time point).

For the differentiation assay, the cells were seeded at 25,000 cell/well and cultured as above. The plates were fixed on days 5 and 8 and then immunostained with the anti-myosin heavy chain (MHC) and anti-AChR antibodies (Supplementary Table S1). The samples were imaged with an Olympus IX83 inverted fluorescent microscope and quantified using the Fiji/ImageJ software (Schindelin et al., 2012) (n = 6 per group/time point). Three quantitative metrics were used to assess the hMPC differentiation: the percent of nuclei in MHC-positive cells (relative to the total number of nuclei), the relative area occupied by MHC-positive cells (normalized to the well area), and the area of individual MHC-positive cells.

To assess the effect of CNTF and GDNF on the efficiency of AChR cluster formation on the differentiated hMPCs, the following metrics were used: the AChR cluster area percentage (i.e., a fraction of the AChR cluster area on MHC-positive cells relative to the total area of MHC-positive cells), the AChR cluster density (calculated as the number of AChR clusters per μ m^2^ of the MHC-positive cell area), and the number of AChR clusters per MHC-positive cell.

### 2.7 Statistical analysis

Two-way ANOVA or aligned rank transform analysis tests (Wobbrock et al., 2011), as appropriate, were performed in the R statistical environment (R Core Team, 2021). Data were plotted using the ggplot2 library (Wickham, 2016).

## 3 Results

### 3.1 PLGA microsphere-based neurotrophic factor delivery system facilitates neurite growth in an *in vitro* neurite growth assay

We previously developed and optimized a microsphere-based neurotrophic factor delivery system aiming at facilitating the innervation of bioprinted skeletal muscle implants (Mashanov et al., 2020; Poerio et al., 2022). We demonstrated that the PLGA microspheres loaded with two neurotrophic factors–CNTF and GDNF–released the neurotrophic factors in a sustained manner and significantly enhanced the neurite outgrowth when incorporated into an acellular bioink (fibrin hydrogel) (Poerio et al., 2022). Here, we investigate the feasibility of using this neurotrophic factor delivery system to facilitate the innervation of cellularized bioprinted muscle constructs.

To evaluate the capability of promoting innervation *in vitro*, we performed a neurite growth assay by co-culturing the bioprinted muscle construct containing hMPCs with chick embryo DRGs.

**FIGURE 3:**
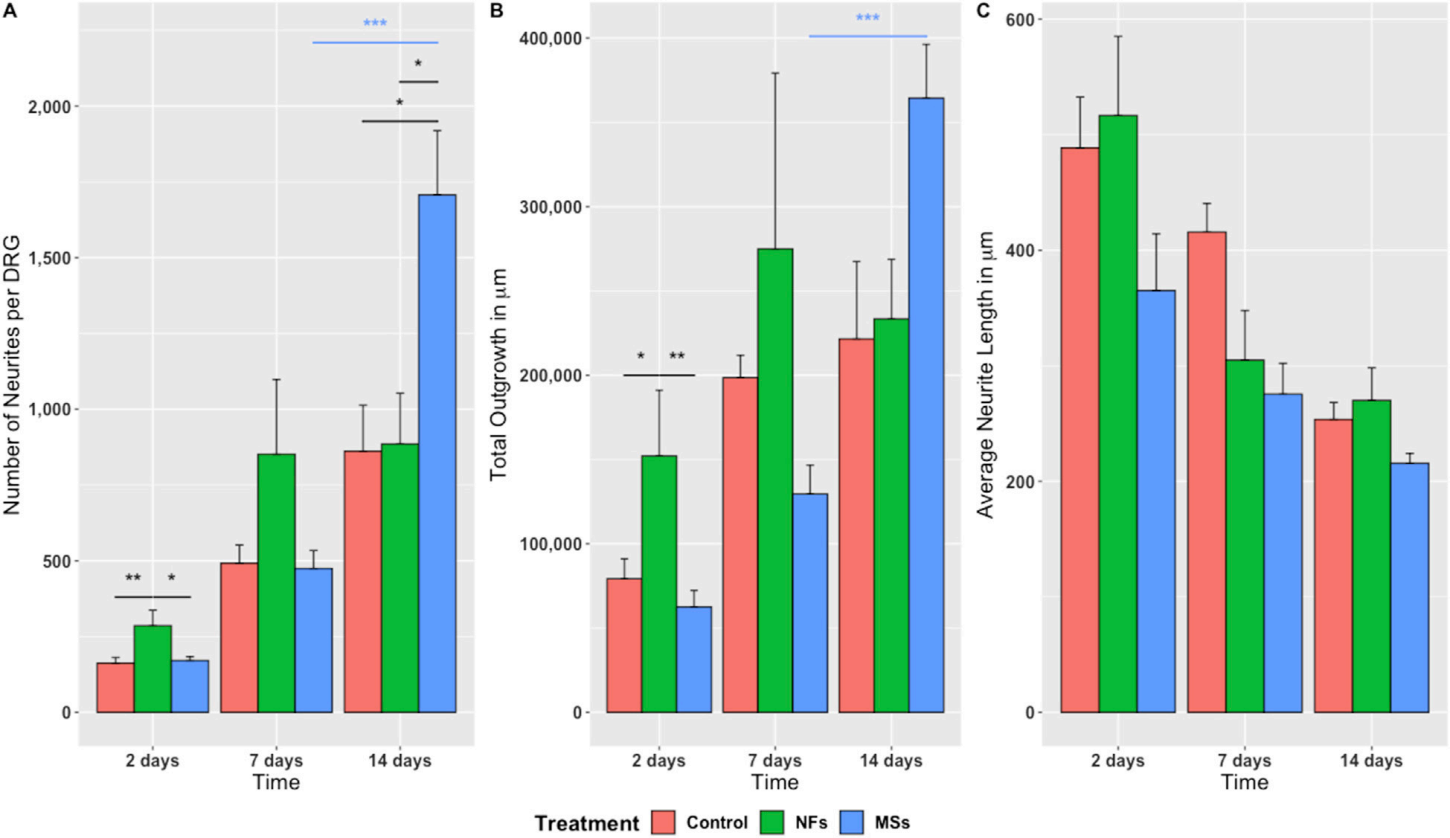
Quantitative assessment of neurite sprouting from the chick embryo DRGs grown on 3D bioprinted skeletal muscle constructs. Three metrics were used: **(A)** Number of neurites sprouting from individual ganglia; **(B)** Total outgrowth (the sum total of neurite length); **(C)** Average neurite length. The significance level annotations are shown in black for the treatment cohorts at each time point and in the respective color for each treatment cohort across time points. Three to eight ganglia were quantified per treatment cohort at each time point. The freely dissolved neurotrophic factors (*NFs*) induced higher neurite sprouting on day 2. This effect was, however, unsustainable. In contrast, the neurotrophic factors delivered in microspheres (*MSs*) induced the sprouting of more neurites per ganglion and higher total neurite outgrowth by day 14. ^*^ p<0.05, ^**^ p<0.01, ^***^ p<0.001 .

The experimental design involved three treatment cohorts: (a) *Control*–bioprinted skeletal muscle constructs containing no extrinsic neurotrophic factors, (b) *NFs*–constructs with CNTF and GDNF directly mixed into the hydrogel, and (c) *MSs*–constructs containing CNTF/GDNF-loaded PLGA microspheres, with the overall load of neurotrophic factors matching that in group (b). The samples were analyzed after 2, 7, and 14 days in culture (Figure 2) using three neurite outgrowth metrics: (1) the number of neurites sprouting from each ganglion (Figure 3A), (2) total neurite outgrowth (Figure 3B), and (3) average neurite length (Figure 3C).

The quantitative statistical analysis detected a significant effect of the treatment on the growth metrics (Supplementary Tables S2–S5). The positive effect of the extrinsic neurotrophic factors was seen in the *NFs* and *MSs* cohorts. However the timing of this effect depended on whether the factors were freely present in the hydrogel or encapsulated in the microspheres. The freely dissolved neurotrophic factors (the *NFs* cohort) induced significantly higher neurite sprouting in terms of neurite number and the total length of neurites per ganglion on day 2 compared to the other two treatment cohorts (Figures 3A, B). This effect tended to persist on day 7, but was no longer sustainable at day 14. In contrast, the CNTF and GDNF delivered in PLGA microspheres (the *MSs* cohort) induced the highest neurite growth over a longer time scale. More specifically, this capacity was manifested in the significantly higher number of neurites (Figure 3A) and total outgrowth (Figure 3B) on day 14, as compared to the other two groups. In addition, the neurotrophic factors released from the microspheres tended to induce more extensive neurite branching, as evidenced by the higher number of neurites (Figure 3A) and higher total outgrowth (Figure 3B) combined with the lower average neurite length (Figure 3C) on day 14 in this cohort.

In addition to the effect of treatment at individual time points, the two-way ANOVA analysis also showed a significant variation in the observed outgrowth parameters over time (Supplementary Tables S2–S5). Importantly, the temporal dynamics were expressed differentially among the treatment cohorts. Only the *MSs* cohort showed a significant increase in neurite number per ganglion and an increase in the total outgrowth during the later stage of the experiment (between day 7 and day 14) (Figures 3A, B).

Taken together, these *in vitro* assay results indicate that the microsphere-based neurotrophic factor delivery system is efficient in supporting long-term neurite outgrowth.

### 3.2 The microsphere-based neurotrophic factor delivery system enhances innervation of biomanufactured skeletal muscle implants in an *in vivo* transposed nerve assay

**FIGURE 4:**
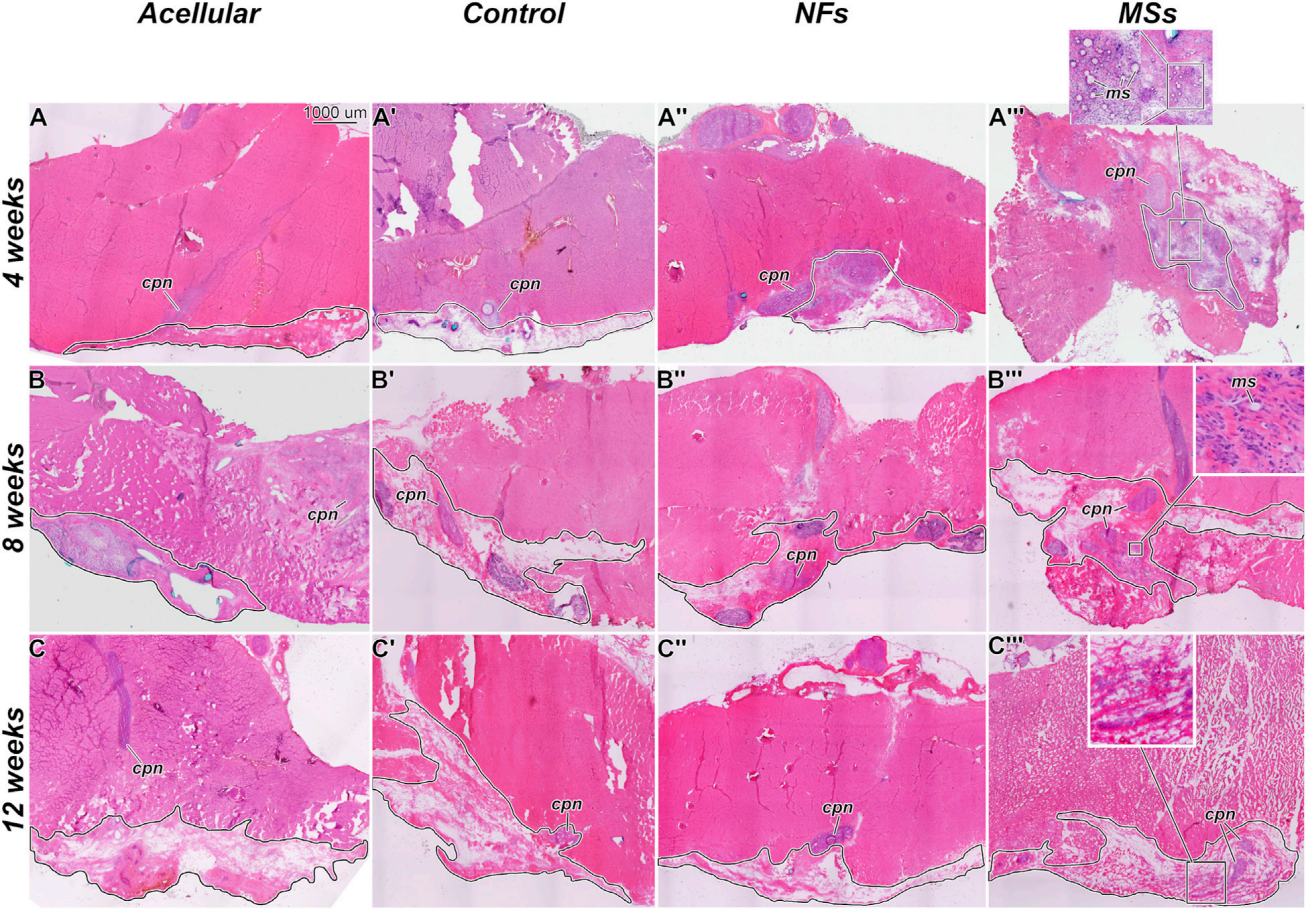
Representative micrographs of the histological organization of the tissue samples from the *in vivo* transposed nerve study at 4 weeks **(A–A‴)**, 8 weeks **(B–B‴)**, and 12 weeks **(C–C‴)** post-implantation. Hematoxylin and eosin staining. The *Acellular* cohort involves fibrin hydrogel implants that contain no cells and no neurotrophic factors. The *Control* group involves biofabricated muscle implants with 30 million hMPCs per mL of hydrogel, but no extrinsic neurotrophic factors. The third group *(NFs*) contains CNTF and GDNF freely dissolved in the extracellular matrix of the implant. The fourth group *(MSs)* contains the matching load of the neurotrophic factors encapsulated in PLGA microspheres suspended throughout the hydrogel of the construct. The implanted constructs are outlined. Note that in the *MSs* cohort, the microspheres are abundant in the extracellular matrix of the implant at 4 weeks post-implantation. After 8 weeks, they become much smaller in size and are no longer detectable after 12 weeks. This indicates that the microspheres slowly dissolve in time and release the encapsulated CNTF and GDNF. *cpn*–transposed common peroneal nerve; *ms*–CNTF/GDNF-loaded PLGA microspheres.

**FIGURE 5:**
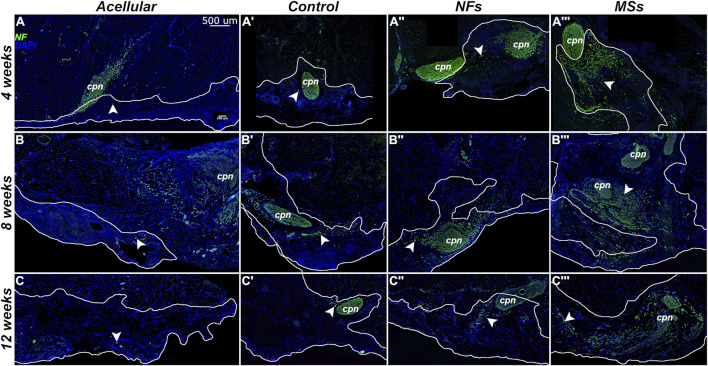
Representative micrographs of the immunostained samples from the transposed nerve study at 4 weeks **(A–A‴)**, 8 weeks **(B–B‴)**, and 12 weeks **(C–C‴)** post-implantation. Sections were immunostained with an anti-neurofilament *(NF)* antibody *(green)* to visualize the sprouting neurites *(arrowheads)*. The implanted constructs are outlined. The nuclei are stained with DAPI *(blue)*. *cpn*–transposed common peroneal nerve.

**FIGURE 6:**
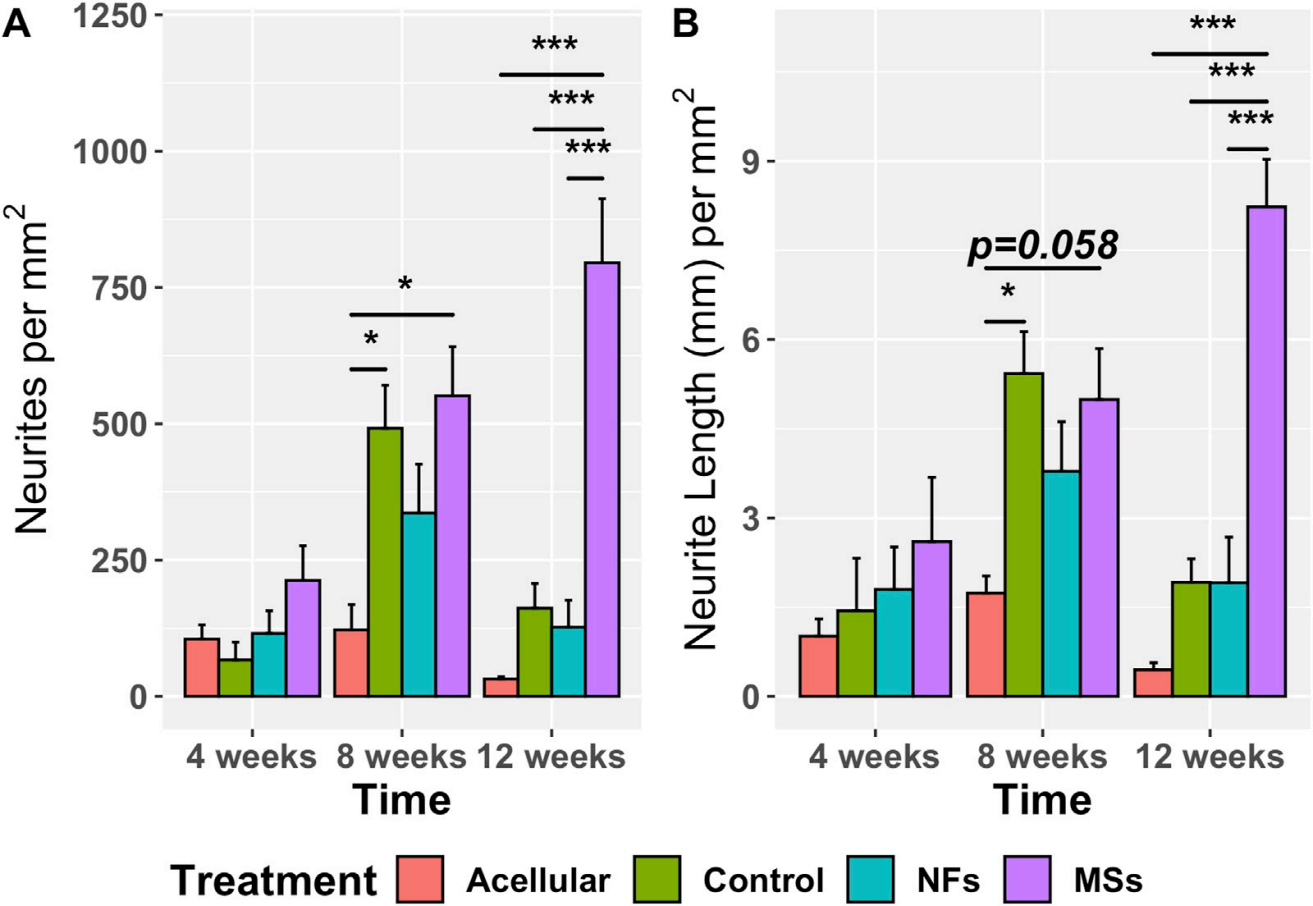
Quantitative assessment of neurite sprouting from the transposed host common peroneal nerve in the implanted skeletal muscle constructs at 4, 8, and 12 weeks post-implantation. The *Acellular* cohort involves fibrin hydrogel implants with no cells and no extrinsic neurotrophic factors. The *Contro*l group contains cellularized biofabricated muscle implants without any extrinsic neurotrophic factors. The third group *(NFs)* contains CNTF and GDNF freely dissolved in the extracellular matrix of the implant. The fourth group *(MSs)* contains the matching load of the neurotrophic factors encapsulated in PLGA microspheres suspended throughout the hydrogel of the construct. There is no statistically significant difference among the treatment cohorts at 4 weeks post-implantation. At 8 weeks, the sprouting in the *Control* and *MSs* cohorts was higher than in the *Acellular* cohort. At 12 weeks, the constructs in the *MSs* cohort showed more extensive sprouting than any of the other three cohorts, indicating that only the CNTF/GDNF delivered in PLGA microspheres were capable of sustaining long-term neurite outgrowth. Three to four animals were analyzed in each treatment group at all three time points. ^*^ p<0.05, ^***^ p<0.001 .

**FIGURE 7:**
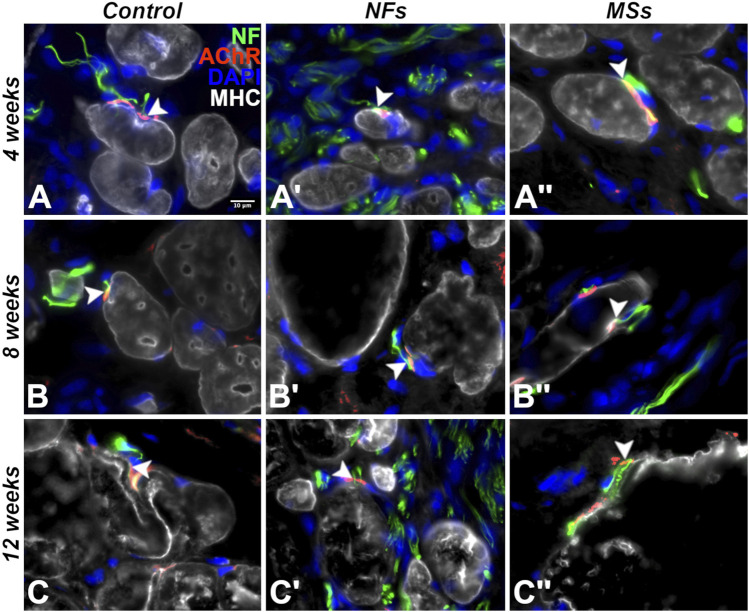
Representative micrographs of differentiation and innervation of skeletal muscle cells in the implants in the *in vivo* transposed nerve study at 4 weeks **(A–A′′)**, 8 weeks **(B–B′′)**, and 12 weeks **(C–C′′)** post-implantation. Triple immunostaining with an AChR antibody *(red)*, an anti-neurofilament *(NF)* antibody *(green)*, and anti-myosin heavy chain (MHC) antibody *(white)*. The nuclei were stained with DAPI *(blue*). *White arrowheads* indicate neuromuscular junctions. As early as 4 weeks post-implantation, the cells in the implant display the proper skeletal muscle cell phenotype and form neuromuscular junctions with the sprouting neurites of the host. The muscle cells in the implant maintained their phenotype and stayed innervated throughout the duration of the experiment (12 weeks post-implantation).

**FIGURE 8:**
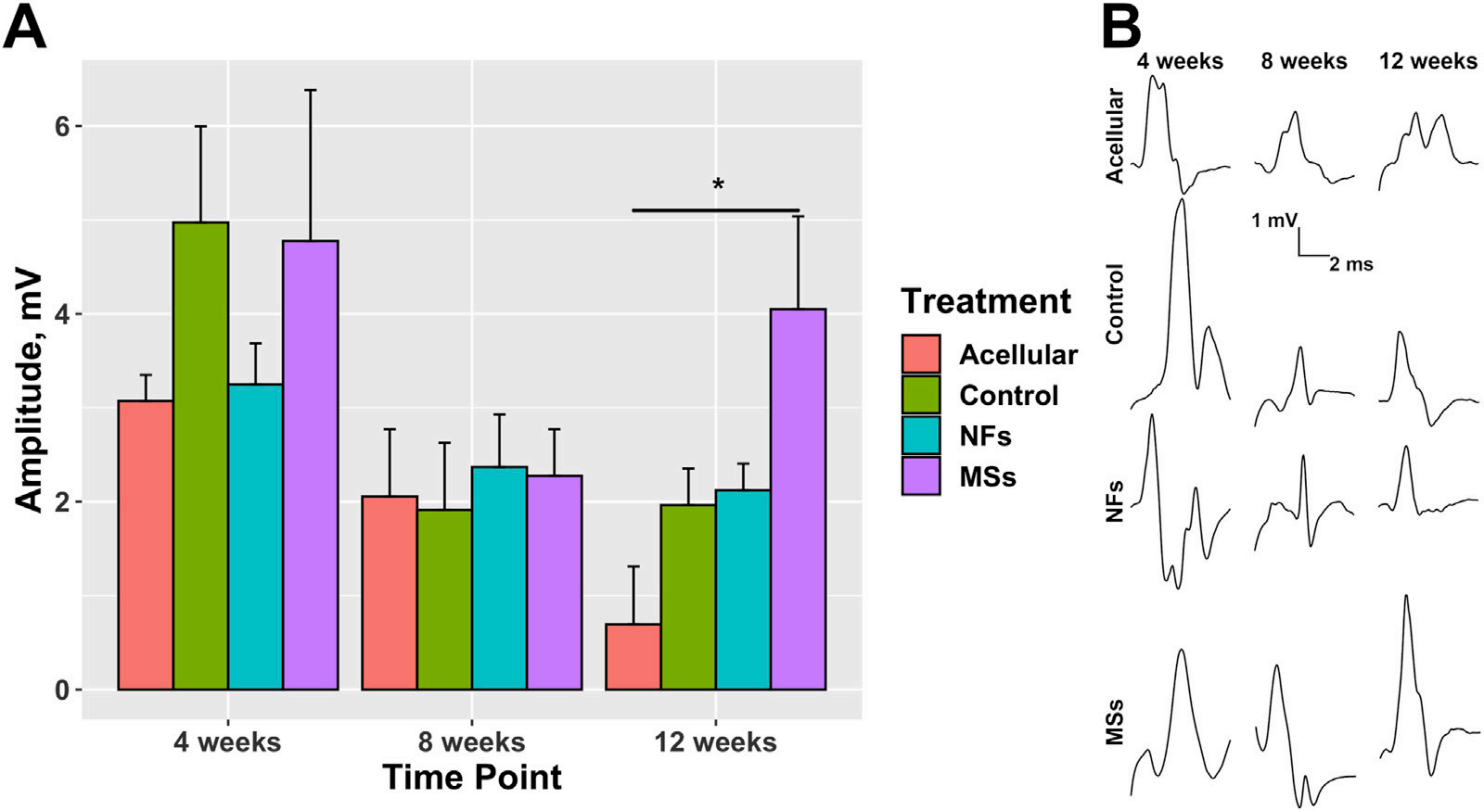
Compound muscle action potential (CMAP) in the transposed nerve model study at 4, 8, and 12 weeks post-implantation. The *Acellular* cohort received bioprinted implants containing only fibrin hydrogel with no cells and no intrinsic neurotrophic factors. The *Control* group contains cellularized biofabricated muscle implants without any extrinsic neurotrophic factors. The third group *(NFs)* contains CNTF and GDNF freely dissolved in the extracellular matrix of the cellularized implant. The fourth group *(MSs)* contains the matching load of the neurotrophic factors encapsulated in PLGA microspheres suspended throughout the extracellular matrix of the construct. **(A)** CMAP amplitude. At 12 weeks post-implantation, the CMAP recorded from the constructs of the *MSs* cohort shows a trend to be higher than in the other two cohorts with cellularized constructs (the *Control* and *NFs* cohorts), and was the only one showing a statistically significant difference from the *Acellular* cohort. These data indicate that the CNTF and GDNF released from the PLGA microspheres facilitate innervation of the skeletal muscle implants after 12 weeks. This effect requires the microsphere-based neurotrophic factor delivery system and cannot be achieved by implementing the neurotrophic factors in the freely dissolved form. Three animals were analyzed in each treatment cohort (n = 3) ^*^ p<0.05 **(B)** Representative waveforms.

To investigate the effect of CNTF and GDNF released from PLGA microspheres on the innervation of the biomanufactured skeletal muscle implants in the *in vivo* settings, we employed a transposed host nerve assay. The distal end of the transected common peroneal nerve (CPN) was placed between layers of the muscle construct, which was implanted under the fascia of the gluteus muscle of athymic rats (Supplementary Figure S2). Four different types of constructs were bioprinted and implanted: (a) acellular constructs without neurotrophic factors (the *Acellular* cohort); (b) cellularized constructs containing hMPCs with preformed AChR clusters, but no extrinsic neurotrophic factors (the *Control* cohort); (c) cellularized constructs with CNTF and GDNF directly dissolved in the hydrogel matrix (the *NFs* group); and (d) cellularized constructs with CNTF and GDNF encapsulated within PLGA microspheres (the *MSs* group). The extent of implant innervation was quantitatively assessed at 4, 8, and 12 weeks post-injury and implantation (Figures 4–8).

The general histological organization of the implants is shown in Figure 4. In the *MSs* cohort implants, the CNTF/GDNF-loaded microspheres remained abundant in the connective tissue matrix of the implant at 4 weeks post-surgery (Figure 4A”’). After 8 weeks, the microspheres were minimally detected, and their size had decreased (Figure 4B”’). At 12 weeks, the microspheres could no longer be detected (Figure 4C”’). These observations indicate that the PLGA microspheres gradually dissolved over time in the *in vivo* environment and released the encapsulated neurotrophic factors.

#### 3.2.1 Neurite sprouting

The effect of the extrinsic CNTF and GDNF on neurite sprouting in the implant was assessed in sections immunostained with an anti-neurofilament antibody (Figure 5). Two metrics were employed (Figure 6): the number of neurites normalized to the implant area and the cumulative length of the sprouting neurites normalized to the implant area.

The omnibus two-way ANOVA assay showed a significant variation in both metrics across the treatment groups over time (Supplementary Tables S6, S7). In the analysis of the simple effect of treatment, *post hoc* Tukey’s test further demonstrated no statistically significant difference in the neurite sprouting metrics among the four treatment cohorts at 4 weeks post-implantation (Figure 6). However, at 8 weeks, the neurite sprouting in the *Control* and *MSs* cohorts was higher than in the *Acellular* cohort. At 12 weeks post-implantation, the constructs with CNTF/GDNF-loaded microspheres (the *MSs* cohort) showed a significantly higher amount of neurite sprouting than any of the other three treatment cohorts.

Furthermore, the neurite sprouting showed different temporal patterns among the treatment cohorts (Figure 6). In the *Acellular* cohort, there was no significant variation over time. In the *Control* and *NFs* cohorts, there was a trend of increasing sprouting between 4 weeks and 8 weeks followed by a decrease at 12 weeks, probably due to neurite pruning, as it commonly happens in neuronal circuitry development (Schuldiner and Yaron, 2015). In contrast, only the *MSs* cohort showed sustainably high levels of neurite sprouting for up to 12 weeks post-implantation.

Taken together, the above observations indicate that: (a) the acellular constructs poorly support neurite outgrowth from the host peripheral nervous system; (b) cellularized constructs of the *Control* cohort support the initial neurite sprouting, but this effect is not sustainable over a longer time span (12 weeks); (c) freely dissolved CNTF and GDNF in the cellularized constructs do not enhance the host neurite sprouting in the implant as compared to the *Control* cohort containing no neurotrophic factors; (d) CNTF/GDNF-loaded microspheres (the *MSs* cohort) facilitate sustained high levels of *in vivo* host neurite outgrowth in the muscle implant over extended periods of time (at least 3 months). The results demonstrate that incorporating the microsphere-based delivery system into the bioprinted muscle constructs is necessary for enhancing the functionality of the neurotrophic factors and subsequent long-term neurite outgrowth *in vivo*.

#### 3.2.2 Innervation of muscle cells in the implant

To assess if the cells in the biomanufactured skeletal muscle implants undergo proper maturation and innervation by the sprouting host neurites, we have performed a series of multi-labeling immunohistochemical assays (Figure 7). As early as 4 weeks post-implantation, many cells within the implant expressed the skeletal muscle differentiation marker sarcomeric myosin heavy chain protein (MHC) (Figure 7A–A”). These cells maintained their phenotype and formed neuromuscular junctions (NMJs) with the sprouting host neurites throughout the duration of the experiment (up to 12 weeks post-implantation) (Figures 7B–C”). The muscle cells in all three cohorts involving cellularized implants (i.e., the *Control*, *NFs*, and *MSs*) consistently formed and maintained neuromuscular junctions, irrespective of the addition of the extrinsic neurotrophic factors. Future studies will explore the potential quantitative impact of extrinsic CNTF and GDNF on the number of synapses formed between the implanted myocytes and host neurites.

To identify human muscle cells within the implant, we performed double immunostaining with a human-specific nuclear antigen antibody and an antibody against the muscle cell differentiation marker desmin (Supplementary Figure S3). Many desmin-positive cells in the implant expressed the human nuclear antigen indicating their derivation from the hMPCs delivered during the surgery (Supplementary Figure S3).

In addition to the histological and immunohistochemical analyses, we performed an electrophysiological test (electromyography/nerve conduction velocity) to assess the level of functional integration of the transposed host nerve into the implanted muscle construct (Figure 8; Supplementary Table S8). At 12 weeks post-implantation, the amplitude of the compound muscle action potential (CMAP) recorded from the implanted constructs containing the neurotrophic factor-loaded microspheres (the *MSs* cohort) showed a trend to be higher than that in the *Control* and *NFs* cohorts. In addition, only the *MSs* exhibited a statistically higher CMAP amplitude compared to the *Acellular* cohort (Figure 8). These physiological data support the conclusions drawn from the morphological analysis and indicate that the PLGA microsphere-based delivery of extrinsic CNTF and GDNF facilitates functional innervation of the skeletal muscle implants. This effect takes up to 12 weeks to develop and cannot be achieved by delivering the neurotrophic factors in the freely dissolved form.

### 3.3 Extrinsic CNTF and GDNF do not impair hMPC viability and differentiation

As described above, we demonstrated that the combination of two neurotrophic factors, CNTF and GDNF, enhances innervation of the biofabricated skeletal muscle implants. However, the effect of these signaling molecules on muscle progenitor cells remains unknown. Thus, we employed an *in vitro* assay to evaluate the effect of CNTF and GDNF on the growth, viability, and differentiation of the hMPCs, as well as their ability to pre-form AChR clusters in response to agrin treatment. The assay involved four cohorts: *Control* (untreated), the *CNTF-only* and *GNDF-only* cohorts, which were treated with individual neurotrophic factors, and the *CNTF + GDNF* cohort, which was treated with a 1:1 combination of the two neurotrophic factors. The concentration of the neurotrophic factors in this *in vitro* assay matched their load established for the biofabrication of the skeletal muscle implant, as described above.

To assess hMPCs growth/viability, an alamarBlue assay was performed on days 0, 3, 5, and 8 (Supplementary Figure S4; Supplementary Table S9). The assay signal steadily increased over time in all four cohorts indicating that the neurotrophic factors, administered either individually or in combination, did not hinder the growth/viability of the hMPCs.

We next assessed the effect of CNTF and GDNF on hMPC differentiation and the agrin-induced AChR cluster formation. Immunocytochemistry with anti-MHC and anti-AChR antibodies was performed on days 5 and 8 (Supplementary Figure S5).

The muscle progenitor cell differentiation was quantified with three metrics: (a) the percent of nuclei in MHC-positive cells (relative to the total number of nuclei), (b) the relative area occupied by MHC-positive cells on the plate, and (c) the size (area) of individual MHC-positive cells (Supplementary Figure S6). Statistical analysis (Supplementary Tables S10–S12) revealed that even though there was a significant increase in the second and third differentiation metrics over time, there were no statistical differences among the treatment cohorts at either of the two time points. These data, therefore, indicate that the neurotrophic factors did not affect the hMPC differentiation.

A similar outcome was seen when we quantified the effect of the neurotrophic factors on the ability of the hMPCs to pre-form AChR clusters in response to agrin treatment (Supplementary Figure S7; Supplementary Tables S13–S15). No differences among the treatment cohorts were observed at the individual time points, except for a minor increase in the number of AChR clusters in the *GDNF* cohort.

In summary, the extrinsic CNTF and GDNF demonstrate no inhibitory effect on hMPC growth/viability, differentiation, or their capacity to pre-form AChR clusters pre-formation. These two neurotrophic factors are therefore suitable for enhancing muscle implant innervation without compromising the functionality of the muscle progenitor cells.

## 4 Discussion

The goal of this study was to develop a clinically applicable strategy for promoting innervation of tissue-engineered skeletal muscle implants by the host’s peripheral axons, thereby facilitating the restoration of damaged muscle function. We previously demonstrated that a specific combination of neurotrophic factors, namely, CNTF and GDNF (in a 1:1 ratio), synergistically induced directed growth of peripheral neurites (Mashanov et al., 2020), indicating that these two factors can serve as chemical cues to boost the natural neural regeneration response and guide the host neurite growth towards the intended targets (muscle cells) within tissue-engineered skeletal muscle implants.

A critical aspect of incorporating signaling molecules into biomanufactured constructs is ensuring that biologically relevant concentrations are maintained postimplantation *in vivo* for a sufficient duration to achieve the desired therapeutic goals (Mohtaram et al., 2013). Given that the regrowth of severed peripheral neurites is a relatively slow process in mammals, which may even decline over time (Chan et al., 2014; Gordon, 2020), the extrinsic neurotrophic factors incorporated into the implants must remain biologically active for at least a few (4–10) weeks (Gordon, 2020). In addition, they need to be protected from dilution and degradation in the *in vivo* milieu (Mohtaram et al., 2013). To address these challenges, we developed a PLGA microsphere-based CNTF/GDNF delivery system (Poerio et al., 2022).

In this study, we demonstrate that the integration of the neurotrophic factor delivery system into bioprinted muscle constructs significantly enhances and sustains neurite sprouting in both *in vitro* and *in vivo* assays. Our findings imply that the sustained release of the neurotrophic factors using the microsphere-based delivery system is necessary for efficient innervation. Indeed, by incorporating the neurotrophic factor delivery system into the muscle implants, we achieved a significant (at least 4-fold) increase in innervation capacity with functional NMJ formation *in vivo*, as demonstrated with immunocytochemistry and physiological recordings.

One limitation of the present study is that the maturation status of neuromuscular junctions newly formed between the sprouting axons of the host and the muscle cells in the implant remains to be further explored at the structural and functional levels. Structurally, techniques such as transmission electron microscopy and combined immunostaining with antibodies against AChR and synaptic vesicle glycoprotein 2 (SV2) can provide valuable insights. Functionally, the synapses can be further assessed with pharmacological perturbation assays and calcium imaging to evaluate synaptic activity.

Achieving proper and timely innervation of muscle implants has remained a major challenge in current tissue engineering-based approaches aimed at treating VML (Ahuja et al., 2021). Denervated muscle cells not only lose functionality due to the lack of nervous system control but also undergo atrophy (Gordon, 2020). A promising strategy pursued thus far involves fabricating composite implants by incorporating neuronal cells into the bioengineered muscle (Das et al., 2020; Kim et al., 2020). While this approach has shown significant improvement in muscle structure and viability, challenges persist, including limited access to autologous neural cell sources and complex protocols for generating implants with multiple cell types. These challenges have hindered clinical applicability and increased manufacturing costs. Moreover, these composite muscle/neuron implants fail to replicate the natural neuromuscular integration and functionality.

Here, we propose a clinically relevant and simplified strategy for bioengineering skeletal muscle constructs aimed at restoring the structure and function of damaged muscles. Our approach involves using the patient’s myocytes as the sole cellular component in the tissue-engineered implants (Ko et al., 2013). The innervation of the cells within the implant, including induced host nerve sprouting, enhanced growth, and functional NMJ formation, is achieved through the incorporation of the delivery system loaded with two defined signaling molecules (CNTF and GDNF). The accelerated innervation of muscle implants translates into improved functional maturation of grafted myocytes, proper control of their physiology by the nervous system, and improved prognosis. This streamlined approach can be further developed into a new generation of clinical strategies addressing extensive injuries of major skeletal muscles in the body. Currently, we have investigated the feasibility of employing this bioprinted muscle construct with the neurotrophic delivery system to restore muscle volume and function with enhanced innervation in an animal VML with a nerve injury model. Additionally, future studies will include in-depth investigations of muscle and nerve regeneration in aspects of physiology and development. The key strategy of this study is to provide an efficient methodology to co-regenerate damaged muscle and peripheral nerve and rebuild denervated muscle-nerve connections (NMJ), all of which are critically required for functional restoration. With further advances, this approach may offer a new therapeutic opportunity for treating severe muscle injuries and, ultimately, enhance the quality of life for patients.

## Data Availability

The original contributions presented in the study are included in the article/Supplementary Material, further inquiries can be directed to the corresponding author.
