# Supplementary material for: Accelerated innervation of biofabricated skeletal muscle implants containing a neurotrophic factor delivery system
Source: Front Bioeng Biotechnol. 2024 Oct 28;12:1476370. doi: 10.3389/fbioe.2024.1476370 (PMC11550949; doi:10.3389/fbioe.2024.1476370)
Supplement: Supplementary file 1 [file DataSheet1.PDF]

Supplementary Material

1 SUPPLEMENTARY FIGURES AND TABLES

1.1 Figures

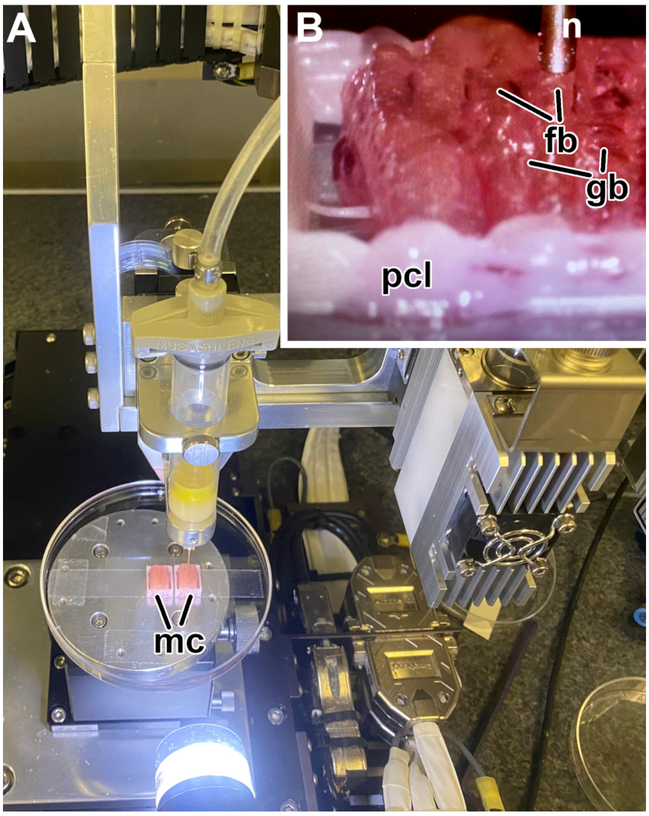

Figure S1. Biomanufacturing of skeletal muscle implants. (A) The 3D Integrated Tissue-Organ Printing (ITOP) system. (B) A skeletal muscle construct in the process of assembly of the second layer. Fibrinogen-based cell-laden bioink is in the process of being extruded from the nozzle and deposited between the filaments of the sacrificial gelatin-based hydrogel. This design of interspersed cell-laden and sacrificial filaments allows for the creation of microchannels in the construct once the sacrificial hydrogel undergoes rapid dissolution. *fb* – fibrinogen-based bioink; *gb* – gelatin-based sacrificial bioink; *mc* – skeletal muscle construct; *pcl* – the outer supporting pillar structure composed of polycaprolactone.

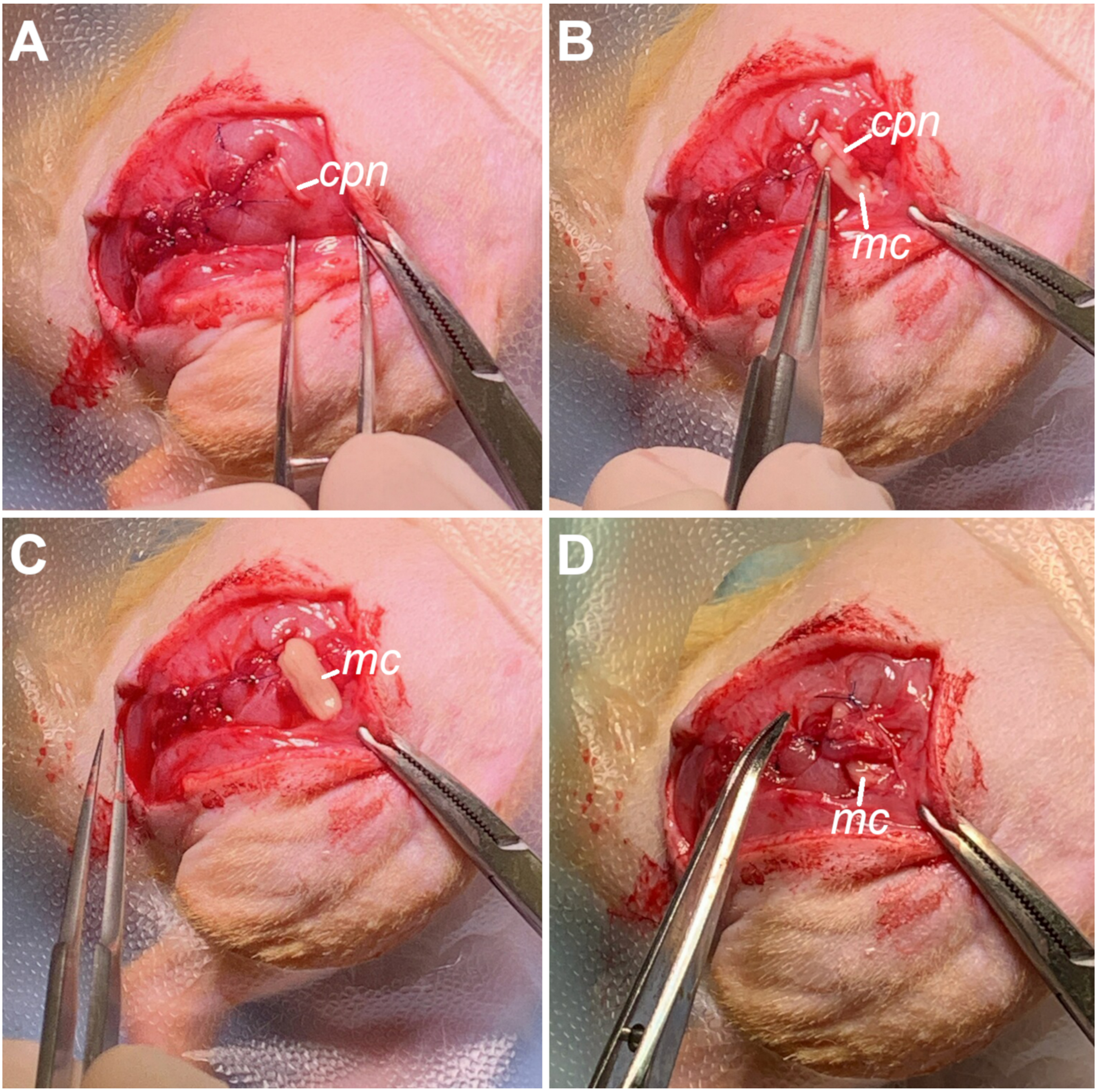

Figure S2. Transposed nerve assay, implantation surgery. (A) The distal end of the common peroneal nerve *(cpn)* is dissected out and placed on the surface of the gluteus muscle. (B) The superficial fascia of the gluteus muscle is opened and one-half of the bioprinted muscle construct *(mc)* is placed on top of the muscle. The distal end of the CPN is placed on top of the construct. (C) The distal end of the CPN is overlaid by the second half of the construct so that the nerve is sandwiched between the two layers of the bioprinted muscle construct. (D) The implanted construct is covered with the fascia, which is then sutured in place.

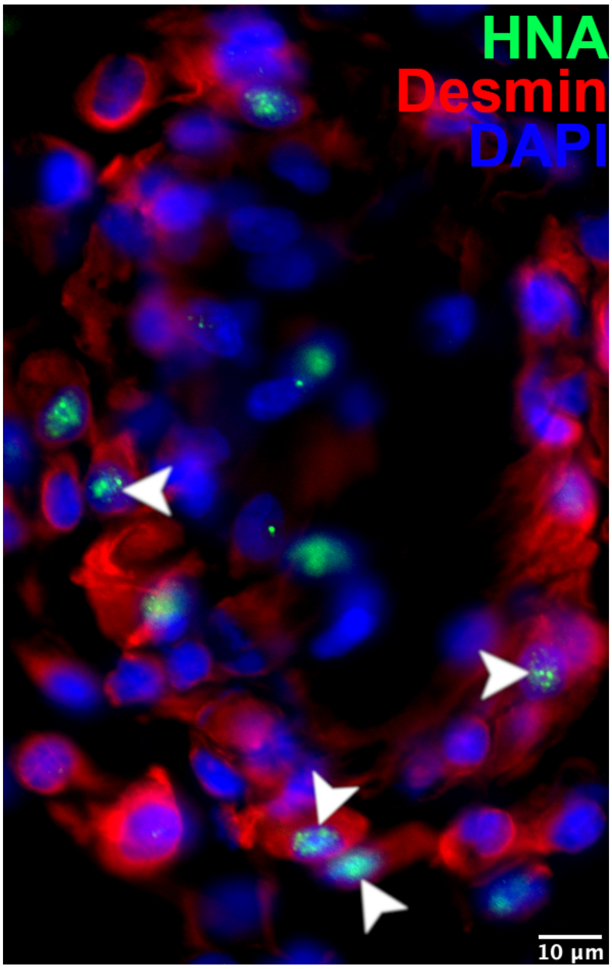

Figure S3. Representative micrograph showing that the implants in the *in vivo* transposed nerve study contains a mix of implanted human cells and infiltrated cells from the adjacent host (rat) muscle at 4 weeks post-implantation. Immunostaining with a human nuclear antigen (HNA) antibody *(green)* and anti-desmin antibody *(red)*. The nuclei were stained with DAPI *(blue)*. The HNA-positive nuclei are indicated by *arrowheads*.

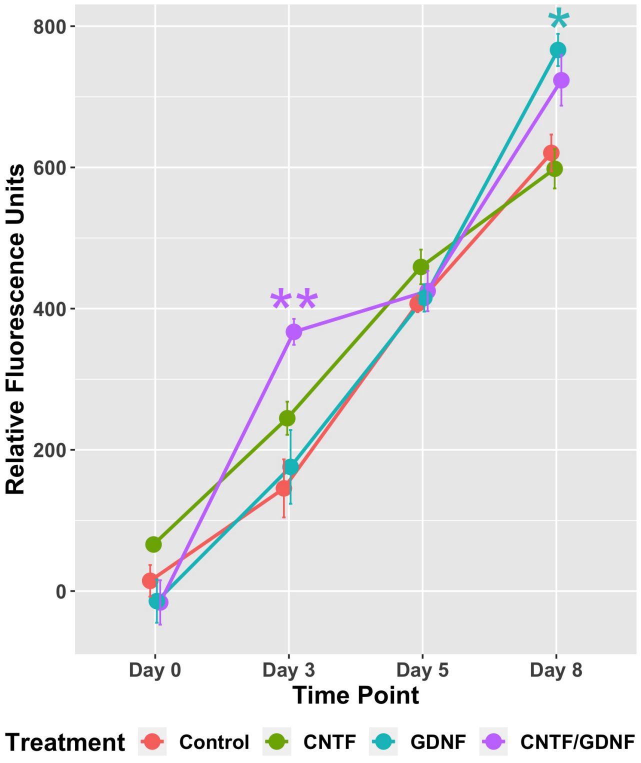

Figure S4. AlamarBlue cell growth/viability assay. hMPCs were incubated in 2D *in vitro* culture in either the base Differentiation Medium *(Control)* or in the same medium containing CNTF and/or GDNF at 0.5 μg/mL. The neurotrophic factors, administered either individually or in combination, did not hinder the growth/viability of the hMPCs, as there was either no statistical difference between the neurotrophic factor-treated and control cohorts, or a slight increase in growth/viability, as in the case of the GDNF-only cohort. Asterisks indicate statistically significant difference from the *Control* cohort: $^{*}p < 0.05$; $^{**}p < 0.01$

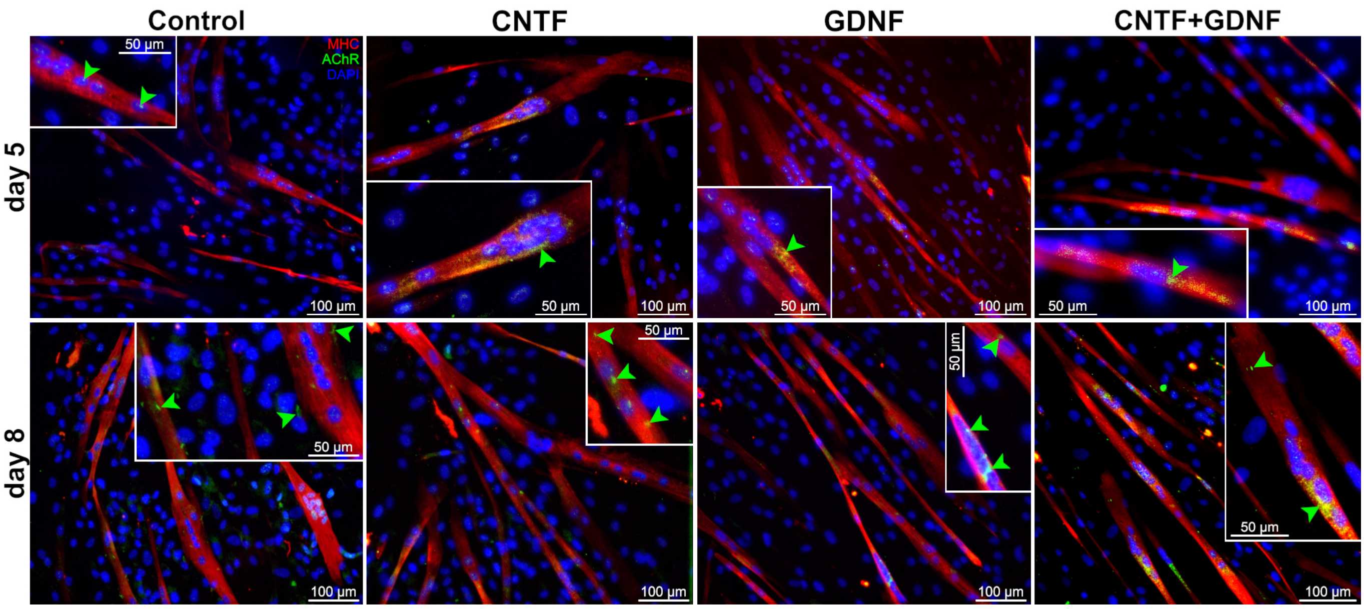

Figure S5. Representative images of hMPCs incubated in 2D *in vitro* culture in either the base Differentiation Medium (Control) or in the same medium containing CNTF and/or GDNF at 0.5 μg/mL. The cells were fixed on day 5 and day 8 and immunostained with antibodies against myosin heavy chain *(MHC, red)* and acetylcholine receptors *(AChR, green)*. Green arrowheads indicate developing AChR clusters

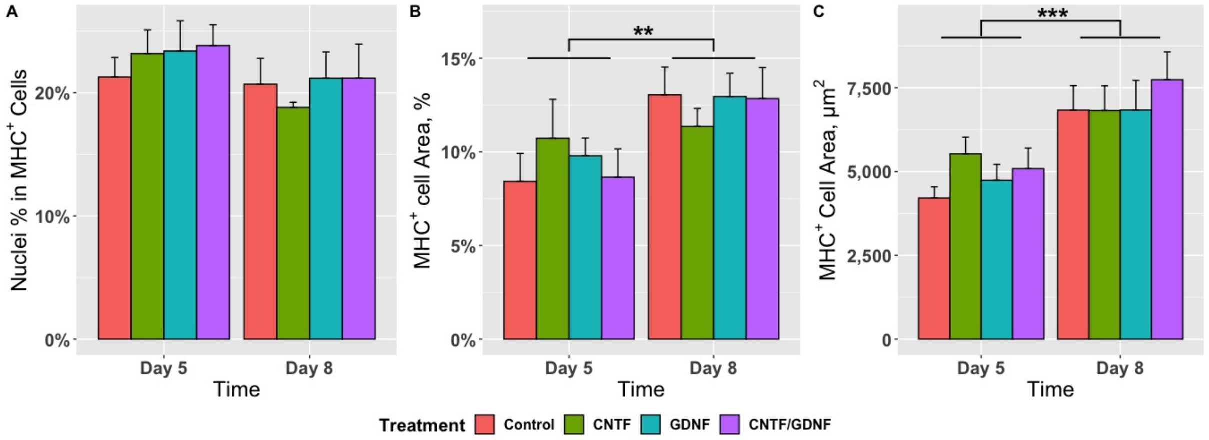

Figure S6. hMPC differentiation metrics in response to treatment with CNTF and/or GDNF at 0.5 μg/mL for 5 or 8 days in an *in vitro* assay. (A) Percentage of nuclei contained in MHC-positive cells; (B) Cumulative area occupied by MHC-positive cells relative to the total area of the field of view; (C) Size (area) of individual MHC-positive cells. Even though there is a significant increase in the second and third differentiation metrics over time, there were no statistical differences among the treatment cohorts at either of the two time points. These data, therefore, indicate that the neurotrophic factors CNTF and GDNF, chosen to facilitate the innervation of biomanufactured muscle constructs, did not affect the differentiation of the hMPCs. $^{**}p < 0.01$; $^{***}p < 0.001$

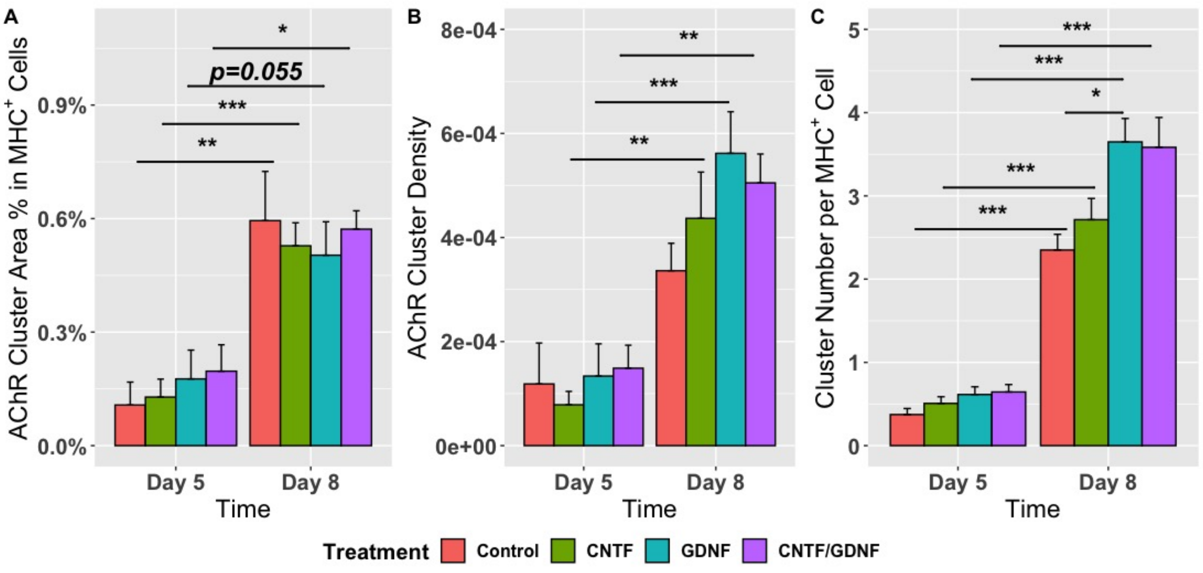

Figure S7. AChR cluster formation metrics in response to treatment with CNTF and/or GDNF at 0.5 μg/mL for 5 or 8 days in an *in vitro* hMPC differentiation assay. (A) AChR cluster area percentage in MHC-positive cells; (B) AChR cluster density in MHC-positive cells; (C) Number of AChR clusters in individual MHC-positive cells. Statistical analysis showed a significant increase in all three metrics in the treatment cohorts over time, but no differences among the treatment groups at individual time points, except for a minor increase in the third metric in the *GDNF* cohort relative to the *Control* cohort on day 8. The data suggest that CNTF and GDNF did not hinder the ability of the hMPCs to pre-form AChR clusters in the presence of agrin. $^{*}p < 0.05$; $^{**}p < 0.01$; $^{***}p < 0.001$

1.2 Tables

Table S1. Antibodies used in this study

Antibody	Vendor (Catalog number)	Host species	Dilution
**Primary antibodies:**			
Neurofilament heavy polypeptide	Abcam (ab4680)	Chicken	1:1,000
Myosin heavy chain	Developmental Studies Hybridoma Bank (MF20)	Mouse	1:50
Human nuclear antigen	Millipore Sigma (MAB1281)	Mouse	1:200
Nicotinic acetylcholine receptor	Millipore Sigma (M217)	Rat	1:1,000
Desmin	Abcam (ab32362)	Rabbit	1:200
**Secondary antibodies:**			
Anti-chicken Alexa Fluor 488	ThermoFisher (A-11039)	Goat	1:200
Anti-chicken Alexa Fluor 647	ThermoFisher (A-21449)	Goat	1:200
Anti-mouse Alexa Fluor 594	ThermoFisher (A-11020)	Goat	1:200
Anti-mouse Alexa Fluor Cy5	ThermoFisher (A10524)	Goat	1:200
Anti-Rat Alexa Fluor 594	ThermoFisher (A-11007)	Goat	1:200
Anti-Rabbit Alexa Fluor 488	ThermoFisher (A-11008)	Goat	1:200

Table S2. *In vitro* neurite outgrowth assay. Number of neurites per ganglion. ANOVA summary table. *Df* – degrees of freedom; *Sum Sq* – sums of squares; *Mean Sq* – mean squares.

	Df	Sum Sq	Mean Sq	F-value	P-value
Treatment	2	472671.92	236335.96	6.70	3.62×10^{-3}
Time Point	2	5469825.89	2734912.94	77.52	3.39×10^{-13}
Treatment:Time Point	4	1547785.50	386946.37	10.97	9.08×10^{-6}
Residuals	33	1164199.17	35278.76		

Table S3. *In vitro* neurite outgrowth assay. Total outgrowth. ANOVA summary table.

	Df	Sum Sq	Mean Sq	F-value	P-value
Treatment	2	44113078578.75	22056539289.38	5.77	7.10×10^{-3}
Time Point	2	209108429924.15	104554214962.07	27.35	9.9×10^{-8}
Treatment:Time Point	4	80612032005.09	20153008001.27	5.27	2.14×10^{-3}
Residuals	33	126139707755.12	3822415386.52		

Table S4. *In vitro* neurite outgrowth assay. Average neurite length. ANOVA summary table.

	Df	Sum Sq	Mean Sq	F-value	P-value
Treatment	2	108440.77	54220.38	5.54	8.41×10^{-3}
Time Point	2	254409.24	127204.62	13.00	6.86×10^{-5}
Treatment:Time Point	4	31326.20	7831.55	0.80	5.34×10^{-1}
Residuals	33	322876.40	9784.13		

Table S5. *In vitro* neurite outgrowth assay. Longest neurite. ANOVA summary table.

	Df	Sum Sq	Mean Sq	F-value	P-value
Treatment	2	2629078.03	1314539.02	2.83	7.34×10^{-2}
Time Point	2	14096144.02	7048072.01	15.17	2.13×10^{-5}
Treatment:Time Point	4	4975875.59	1243968.90	2.68	4.88×10^{-2}
Residuals	33	15330264.89	464553.48		

Table S6. *In vivo* transposed nerve assay. Neurite number. ANOVA summary table.

	Df	Sum Sq	Mean Sq	F-value	P-value
Treatment	3	936067.38	312022.46	19.81	1.92×10^{-7}
Time Point	2	532793.43	266396.71	16.91	9.73×10^{-6}
Treatment Time Point	6	604679.06	100779.84	6.40	1.67×10^{-4}
Residuals	32	504036.13	15751.13		

Table S7. *In vivo* transposed nerve assay. Cumulative outgrowth. ANOVA summary table.

	Df	Sum Sq	Mean Sq	F-value	P-value
Treatment	3	80.90	26.97	13.00	1.01×10^{-5}
Time Point	2	42.51	21.25	10.25	3.63×10^{-4}
Treatment:Time Point	6	62.19	10.37	5.00	1.032×10^{-3}
Residuals	32	66.37	2.07		

Table S8. Amplitude of the CMAP in the transposed nerve model study. ANOVA summary table.

	Df	Sum Sq	Mean Sq	F-value	P-value
Treatment	3	14.58	4.86	2.75	6.49×10^{-2}
Time Point	2	27.04	13.52	7.65	2.7×10^{-3}
Treatment:Time Point	6	11.99	2.00	1.13	3.75×10^{-1}
Residuals	24	42.42	1.77		

Table S9. AlamarBlue cell growth/viability assay. ANOVA summary table.

	Df	Sum Sq	Mean Sq	F-value	P-value
Treatment	3	73672.88	24557.63	5.05	2.96×10^{-3}
Time	3	5752999.49	1917666.50	394.27	$<2 \times 10^{-16}$
Treatment:Time	9	253828.81	28203.20	5.80	3.52×10^{-6}
Residuals	80	389111.76	4863.90		

Table S10. hMPC differentiation in response to treatment with CNTF and/or GDNF. Percentage of nuclei contained in MHC^{+}-cells. ANOVA summary table.

	Df	Sum Sq	Mean Sq	F-value	P-value
Treatment	3	0.00	0.00	0.33	0.8042
Time	1	0.01	0.01	3.17	0.0830
Treatment:Time	3	0.00	0.00	0.29	0.8344
Residuals	38	0.09	0.00		

Table S11. hMPC differentiation in response to treatment with CNTF and/or GDNF. Cumulative area occupied by MHC^{+}-cells relative to the total area of the field of view. ANOVA summary table.

	Df	Sum Sq	Mean Sq	F-value	P-value
Treatment	3	0.00	0.00	0.08	0.9679
Time	1	0.01	0.01	8.87	5.03×10^{-3}
Treatment:Time	3	0.00	0.00	0.75	0.5299
Residuals	38	0.05	0.00		

Table S12. hMPC differentiation in response to treatment with CNTF and/or GDNF. Size (area) of individual MHC^{+}-cells. Aligned rank transform analysis summary table.

	Df	Df.res	Sum Sq	Sum Sq.res	F value	P-value
Treatment	3	1001	92509.69	81800288.55	0.38	0.7694
Time	1	1001	1182217.32	83154173.03	14.23	1.71×10^{-4}
Treatment:Time	3	1001	119798.84	81031969.24	0.49	0.6870

Table S13. AChR cluster formation on muscle cells in response to treatment with CNTF and/or GDNF. AChR cluster area percentage normalized to the area of MHC^{+} cells. ANOVA summary table.

	Df	Sum Sq	Mean Sq	F-value	P-value
Treatment	3	0.00	0.00	0.22	0.8816
Time	1	0.00	0.00	55.65	5.92×10^{-9}
Treatment:Time	3	0.00	0.00	0.38	0.7687
Residuals	38	0.00	0.00		

Table S14. AChR cluster formation on muscle cells in response to treatment with CNTF and/or GDNF. AChR cluster density in MHC^{+} cells (Number of cluster per $1\mu m^2$ of MHC^{+} cell area). ANOVA summary table.

	Df	Sum Sq	Mean Sq	F-value	P-value
Treatment	3	0.00	0.00	1.53	0.2231
Time	1	0.00	0.00	58.28	3.47×10^{-9}
Treatment:Time	3	0.00	0.00	0.88	0.4601
Residuals	38	0.00	0.00		

Table S15. hMPC differentiation in response to treatment with CNTF and/or GDNF. Number of AChR clusters per MHC^{+}-cell. Aligned rank transform analysis summary table.

	Df	Df.res	Sum Sq	Sum Sq.res	F value	P-value
Treatment	3	1017	4046862.53	79958789.28	17.16	7.11×10^{-11}
Time	1	1017	29644990.99	57708912.93	522.43	2.22×10^{-16}
Treatment:Time	3	1017	1906103.31	82088321.86	7.87	3.40×10^{-5}
